# 

**Published:** 1885-02

**Authors:** 


### BUREAU OF CLINICAL MEDICINE: I. H. A.

Meeting to be held at Syracuse, June 23d, 1885.

P. P. Wells, M. D., Brooklyn, paper on "Clinical Duties;"
John F. Miller, M. D., three cases of Diabetes cured;
E. Rushmore, M. D., Plainfield, N. J., Clinical cases;
Frederick Ehrman, M. D., Cincinnati, cases of Arthritis;
Samuel Swan, M. D., New York, three cases of Headache;
Mahlon Preston, M. D., cases of Phthisis pulmo.;
J. F. Bigler, M. D., Rochester, Clinical cases;
C. Pearson, M. D., the "Care of the Stomach in Clinical Cases."

Papers may also be expected from E. W. Berridge, M. D., of England; A. Charge, M. D., Toulon, France; Dr. Alvarez, Madrid, Spain, and others. C. PEARSON, Chairman.

